# Moxibustion for treating chronic pelvic inflammatory disease: A protocol for systematic review and meta-analysis

**DOI:** 10.1097/MD.0000000000021925

**Published:** 2020-08-28

**Authors:** Fanghui Hua, Honglian Li, Jun Xiong, Shouqiang Huang, Jie Xiang, Xiaohong Zhou

**Affiliations:** a Jiangxi University of Traditional Chinese Medicine, Nanchang; b Haiyang people's Hospital of Shandong Province, Haiyang; c The Affiliated Hospital of Jiangxi University of Traditional Chinese Medicine, Nanchang, China.

**Keywords:** chronic pelvic inflammatory disease, moxibustion, protocol, systematic reviews

## Abstract

**Background:**

Chronic pelvic inflammatory disease (CPID) is a difficult-to-treat gynaecological disorder, which has complex etiologies, among married women. In recent years, moxibustion has gradually shown its clinical advantages and been more and more widely used In China. The protocol is try to synthesize and assess the effectiveness and safety of moxibustion for patients with CPID.

**Methods:**

Seven databases as following: PubMed, Embase, Cochrane Library, China National Knowledge Infrastructure, WangFang Database, Chinese Scientific Journal Database, Chinese Biomedical Literatures Database will be searched from their inception to May 2020. No restrictions about language and status. Study selection, data collection, and quality assessment will be respectively conducted by 2 researchers. Based on the heterogeneity test results, the fixed-effects or random-effects model will be selected to synthesize data. The effective rate, Pelvic inflammatory mass diameter and Pelvic fluid depth will be the primary outcomes. Patient reported outcome scale, visual analog scale, C-reactive protein, transforming growth factor β1 =  transforming growth factor β, incidence of any adverse events will be the secondary outcomes. Revman 5.4 software will be implemented for data synthesis. Dichotomous data will be represented by risk ratio for efficacy and safety of CPID treated with moxibustion, while continuous data will be represented by mean difference with a 95% confidence interval.

**Results:**

The results of this study will be published in a peer-reviewed journal. This study will provide a comprehensive review of the available evidence for the treatment of moxibustion with CPID.

**Conclusions:**

This study expects to provide high-quality, evidence-based recommendations on further treatment for clinical guidance of CPID.

**Trial registration number:**

CRD42020158744 in PROSPERO 2020.

## 1 Introduction

Chronic pelvic inflammatory disease (CPID)^[1]^ is common dysfunction characterized by lower urinary tract symptoms and abdominal pain along with irregular menstruation, dysmenorrhea, which aggravating after overwork or sexual intercourse. It is a leading cause of infertility and ectopic pregnancy, and is related to an increased risk of ovarian tumor.^[2]^

At present, the global incidence of PID is about 2% ∼ 12%,^[3]^ and the regional hospitalization rate is about 63.3/100,000 (95% confidence interval [CI] 60.8 ∼ 65.9).^[4]^ At the same time, 18% of PID patients may have chronic pelvic pain, and 10% ∼ 20% may have secondary infertility, which seriously affects the reproductive health and quality of life of women of childbearing age.^[5,6]^ The incidence of pelvic infectious disease is 4.4% among sexually active women aged 18 to 44 years in the United States.^[7]^ In the UK, Chronic pelvic pain costs an estimated £158 million/year to the NHS.^[8]^ Studies have shown that the incidence of PID among women of childbearing age in developing countries is as high as 40%.^[9]^ The average annual medical expenditure per case is about $3,025 in the US^[10]^ and about £163 per patient in Hong Kong.^[11]^

The etiological factors are complex, which include polymicrobial infection, pelvic musculoskeletal disorders, and psychoneurological causes.^[12]^ It results from bacterial infection including chlamydia trachomatis, neisseria and genital mycoplasmas with many predisposing factors including instrumentation of the female genital tract such as uterine curettage, insertion of intrauterine contraceptive devices and hysterosalpingography and vaginal douching, or secondary to adjacent inflammation as appendicitis or some distant focus as pulmonary tuberculosis.^[13]^ Chlamydia trachomatis is the most common cause of the pelvic inflammatory disease (PID) in the United States.^[14]^

Western medicine in the treatment of CPID is given priority to antibiotic treatment against infection, and surgical treatment when necessary. However, as the course deferment, illness becomes increasingly complex, of which often by a variety of pathogenic bacteria mixed infection of the disease. Combination of broad-spectrum antibiotics is not targeted therapy, so the effect is not very ideal. What's more, using antibiotics in large doses over long periods leads to a double infection, as well as the resistance easily.^[15]^ In the case of surgery, the patients need surgical indications, which are usually found to be resistant to antibiotics. Besides, surgery may cause iatrogenic uterine damage.^[16]^ As a result, many patients with CPID migrate from the above therapy and seek alternatives which are effective and safe.

Pelvic inflammation is unrecorded in ancient Books of Traditional Chinese medicine, and its clinical characteristics lump it as “heat discharge into the blood chamber”, “abdominal pain”, “carrying the disease”, “postpartum fever”, “infertility”.^[17]^ It is explained that chronic pelvic inflammation is mostly caused by an invasion of pathogenic heat or dampness and blood circulation disorder leading to lower abdominal pain and other symptoms according to the TCM theory of differential diagnosis and treatment.^[18]^ The pathogeny of it includes qi stagnation and blood stasis staying in Chong Meridian, Ren Meridian, uterus, and uterine collateral, which are mostly caused by the evil invasion of heat-dampness and cold-dampness, liver stagnation and kidney-Yang deficiency.^[19]^ Moxibustion is a TCM therapy for some chronic and debilitating diseases, which igniting the heat of moxa to stimulate the acupuncture points. It stimulates the blood circulation through the warm, promotes the inflammation to subside, and has the effect of bidirectional regulation on the body function state.^[20]^ At present, it has been found that moxibustion can adjust the function of zangfu, promote metabolism, change the amount of blood composition, increase the phagocytosis of red blood cells, white blood cells, haemoglobin and white blood cells, enhance immunity and improve health.^[21]^

Nowadays, a large number of clinical studies in moxibustion regulating CPID have been carried out. Nevertheless, no systematic reviews has reported the effectiveness of moxibustion on CPID. Thus, we conducted this protocol of moxibustion as an intervention for CPID patients in order to achieve a better therapeutic effect.

## 2 Objectives

This study is aimed at summarizing the clinical evidence about the effectiveness and safety of moxibustion for CPID and provide doctors, patients, policy decision-makers with reliable recommendations.

## 3 Methods and analysis

### 3.1 Study registration

This protocol report is structured according to the Preferred Reporting Items for Systematic Reviews and Meta-analysis Protocols in the Cochrane Handbook.^[22]^ It is registered on the International Prospective Register of Systematic Reviews (PROSPERO no. CRD42020158744; in PROSPERO 2020; Available at: http://www.crd.york.ac.uk/PROSPERO/display_record.php?ID=CRD42020158744.

Ethics approval is not required for this review as we will analyze published literature only.

### 3.2 Inclusion certain for study selection

#### 3.2.1 Types of studies

Without restrictions on language and publication status, all the randomized controlled trials (RCTs), which is stated the “randomization” phrase and without blinding restriction, will be included. The animal mechanism studies, case reports, or non-RCTs are excluded.

#### 3.2.2 Types of participants

Patients diagnosed as CPID regardless of race, age, status or education, marital status, and economic status. Trials will be included which applying validated diagnosed criteria, for instance, WHO/NIH/OSHK guideline, Chinese guidelines for diagnosis, and treatment of CPID.

#### 3.2.3 Types of interventions

RTCs that involved any form of moxibustion (eg, direct moxibustion, indirect moxibustion, heat-sensitive moxibustion, moxa burner moxibustion, warm-needling, crude drug moxibustion, or natural moxibustion) as the sole treatment or as a part of combination therapy with another intervention (eg, conventional drugs) will be included. While combined with any complementary therapy will be excluded, for example, Chinese herb decoction, acupuncture, and other complementary therapy.

#### 3.2.4 Type of comparator(s)/control

There is no limit to the treatment of the control group, including no treatment, or placebo, or

The following treatment comparisons will be included in the analysis:

(1) Moxibustion compared with no treatment;
(2) moxibustion compared with placebo or sham moxibustion therapy;
(3) moxibustion compared with other active therapy;
(4) moxibustion plus other active therapy compared with the same active therapy.

#### 3.2.5 Types of outcome measures

##### 3.2.5.1 Primary outcomes

The effective rate, pelvic inflammatory mass diameter and Pelvic fluid depth.

##### 3.2.5.2 Secondary outcomes

Secondary outcome measures will include:

(1) Patient reported outcome scale
(2) Visual analog scale.
(3) C-reactive protein.
(4) transforming growth factor β1 =  transforming growth factor β1.
(5) incidence of any adverse events

### 3.3 Exclusion certain for study selection

The exclusion certain contain the following items:

(1) Patients with acute medical conditions or pregnancy
(2) The literature related to the same study, and duplicated publications
(3) Unable to get literature for available data or full text through various means

### 3.4 Search methods for identification of studies

Seven databases as following: PubMed, Embase, Cochrane Library, China National Knowledge Infrastructure, WangFang Database, Chinese Scientific Journal Database, Chinese Biomedical Literatures Database will be searched from their inception to May 2020. To describe moxibustion for CPID, the initial search strategy (Table 1) was conducted for the PubMed database using MeSH items and free words. Search strategies for the other databases will be adapted as necessary. The following search terms will be used: CPID, chronic pelvic inflammation, chronic pelvic infection, chronic endometritis, sequelae of pelvic inflammatory disease, chronic salpingitis, chronic parametritis, moxibustion, thunder fire miraculous moxa roll, thunder fire moxibustion, taiyi miraculous moxa roll, suspended moxibustion, mild moxibustion, needle warming moxibustion, randomized controlled trial, controlled clinical trial, randomized etc.

**Table 1:**
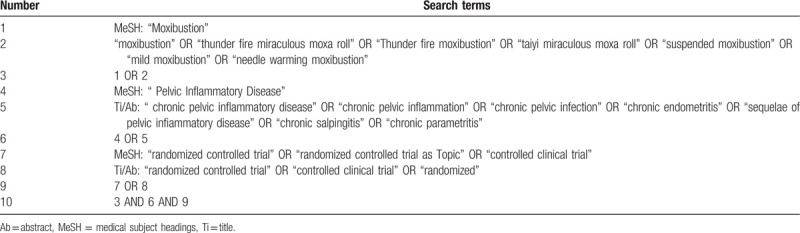
The search strategy for Pubmed.

Also, we will search for eligible trials, which are ongoing or unpublished, in the WHO International Clinical Trial Registration Platform and the China Clinical Trial Registration Platform to make a comprehensive literature search.

### 3.5 Data collection and analysis

#### 3.5.1 Pilot search

To better understand the inclusion and exclusion criteria of each systematic review, we will conduct a pre-test, which is to randomly select some literature for screening. As for the differences of literature screening results, we will exchange and discuss to make the future literature selection more uniform.

#### 3.5.2 Selection of studies

**Figure 1:**
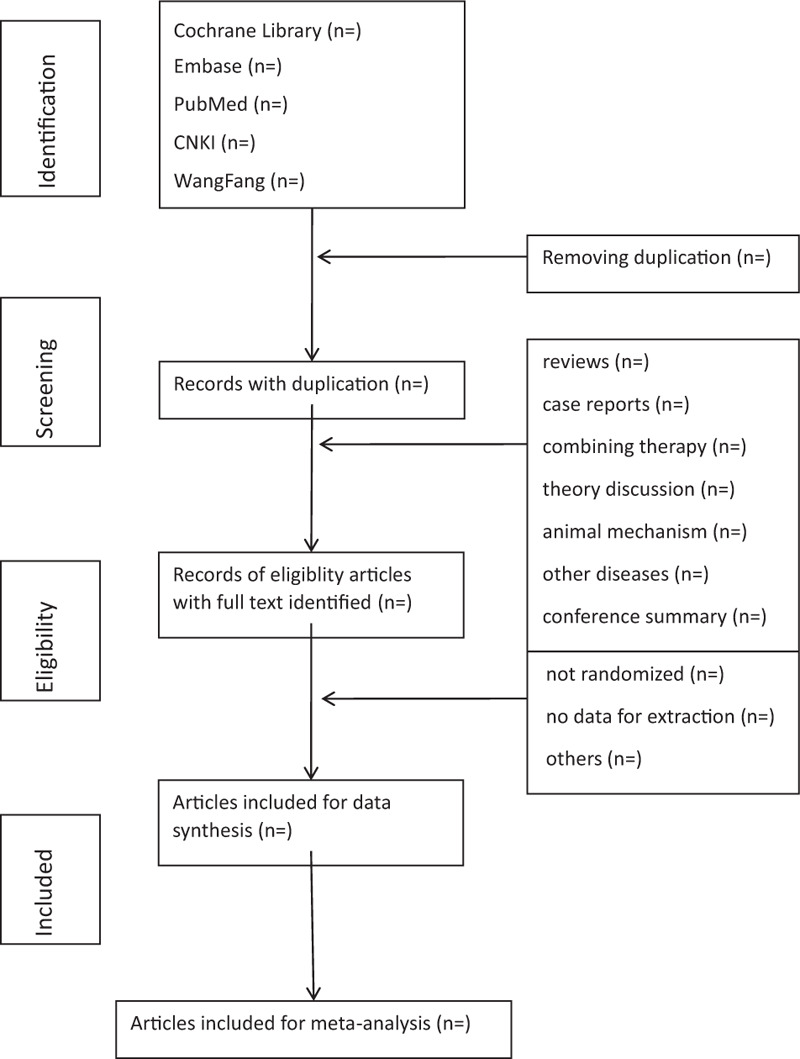
Flowchart of literature selection.

Each literature of title and abstract is scanned by 2 reviewers (QSH and JX) respectively to eliminate some ineligible articles. Then all relevant articles of full text are investigated. When the 2 reviewers cannot agree on the selection process through consultations, the third reviewer (XHZ) will ultimately make the decision. If there is an article with unclear information or missing data, HFH will attempt to contact the original author for the full text and data. The primary selection process is shown in a Preferred Reporting Items for Systematic Reviews and Meta-Analyses flow chart (Fig. 1).

#### 3.5.3 Data extraction

Two independent reviewers (QSH and HFH) shall design a standardized data extraction form, which will be performed by Microsoft Excel, independently extract data and cross-check. The extracted information includes descriptions of studies, characteristics of participants, interventions of both the observation group and control group, quality, randomization, allocation concealment and blinding methods, outcome measures, primary outcomes, adverse effects, duration of follow-up, type and source of financial support, and the Standards for Reporting Interventions in Controlled Trials of Moxibustion (STRICTM) checklist. In case of any discrepancies, we will resolve by discussion and reach a consensus, and if necessary, seek arbitration from a third reviewer (JX). For RCTs lacking important information or data, the reviewers will track back to the primary studies of them.

### 3.6 Assessment of risk of bias and reporting of study quality

The assessment will be conducted by 2 reviewers (QSH and JX) with the risk-of-bias assessment method from Cochrane Reviewer's Handbook 5.0.24,^[23]^ the Consolidated Standards of Reporting Trials^[24]^ and Standards for Reporting Interventions in Clinical Trials of Moxibustion checklist.^[25]^ The risk of bias in included studies will be evaluated according to the seven aspects: randomly generated sequence number, allocation sequence concealment, blinding of participants and personnel, blinding of outcome assessors, incomplete outcome data, selective outcome reporting and other sources of bias. Each of them will be classified into low risk, high risk, and unclear risk based on information provided by the trials. Inconsistency will be consulted with the third review author (HLL). For the data which is missing or ambiguous, we will try to contact the author as possible.

### 3.7 Measures of treatment effect

We will be conduct statistical analyses by RevMan 5.4 software. Weight Mean difference (WMD) with 95% CIs will be used to analyze continuous data. Other forms of data will be changed into WMD values. As for the results of dichotomous data, risk ratio (RR) and its 95% CI will be used.

### 3.8 Data analysis

Data from parallel-group studies will be collected for analysis. If multiple nonmoxibustion control groups are included, pooled analyses of the control groups against the intervention group will be used. We will gather individual data of each result index for evaluation.

### 3.9 Management of missing data

If there are missing or incomplete data for the primary results, we will contact the corresponding authors for the missing data to get specific information by telephone or email. If we can’t obtain that missing information, we will exclude from the analysis.

### 3.10 Assessment of heterogeneity

The difference among studies in the systematic review,^[26]^ which called heterogeneity, is quantified by the value of *I*^2^. The magnitude of heterogeneity was categorized by the *I*^2^ statistic with *I*^2^ of 0% to 24% = no heterogeneity, *I*^2^ of 25% to 49% = moderate heterogeneity, *I*^2^ of 50% to 74% = substantial heterogeneity, and *I*^2^ of 75% to 100% = considerable heterogeneity. When the results are substantial heterogeneity or considerable heterogeneity, sensitivity analysis or subgroup analysis will be made to look for the possible causes.

### 3.11 Assessment of reporting biases

If more than 10 trials are included, the funnel plots will be used to assess reporting biases. If funnel plot asymmetry is detected, the reasons will be analyzed.

### 3.12 Synthesis of data

We will use RevMan for all statistical analyses. There are 2 modes to choose from: the random-effects model (*I*^2^≥50%) or fixed-effects model (*I*^2^ < 50%). If the substantial heterogeneity cannot be identified or there are not enough RCTs, we will perform a narrative, the qualitative summary or subgroup analysis.

### 3.13 Subgroup analysis

If the necessary data are available, subgroup analysis will be carried out according to different factors as follows: the type of CPID, type of moxibustion, the duration or dosage of moxibustion, period of treatment, and the type of intervention in the control group or the study group.

### 3.14 Sensitivity analysis

A sensitivity analysis will be performed when there is significant heterogeneity according to the following aspects: sample size, heterogeneity qualities, methodological elements, and characteristic of research. If heterogeneity is reduced after low-quality or small sample studies are excluded, and we must be more cautious in concluding.

### 3.15 Grading the quality of evidence

The grading of evidence quality will be conducted by 2 independent reviewers using the grading of recommendations, assessment, development and evaluation system instrument.^[27]^ All studies will be rated as 4 levels as following: high, moderate, low or very low, which according to the five aspects (inconsistency, limitations, imprecision, indirectness, and publication bias).^[28]^

## 4 Discussion

CPID is a major gynecological health problem affecting more than 1 million women yearly. It begins with cervicitis and progress leading to serious clinical consequences, which including endometritis, salpingitis, pelvic peritonitis, tubal infertility, ectopic pregnancy, pelvic adhesions, pelvic abscess and chronic pelvic pain.^[29]^ What's more, PID is a risk marker for CHD that is independent of traditional CHD risk factors.^[30]^ Therefore, it is meaningful to resort to complementary approach to manage CPID.

This review, which will be the first summary of existing RTCs of moxibustion treatment of CPID, can help medical workers choose effective and safe interventions. The full discussion section of the report is planned to include the following: main findings, advantages and limitations, comparison with other studies and opinions, interpretation of results and conclusion.

## Author contributions

All authors have read and approved the publication of the protocol.

**Conceptualization:** Fanghui Hua, Jun Xiong.

**Data curation:** Jie Xiang, XiaoHong Zhou, Shouqiang Huang.

**Formal analysis:** Shouqiang Huang, Jie Xiang.

**Investigation:** Jun Xiong, Honglian Li, XiaoHong Zhou.

**Methodology:** Fanghui Hua, Shouqiang Huang, Jie Xiang.

**Software:** XiaoHong Zhou, Honglian Li.

**Supervision:** Jun Xiong, Honglian Li.

**Writing – original draft:** Jun Xiong, Fanghui Hua, Shouqiang Huang, Jie Xiang.

**Writing – review & editing:** XiaoHong Zhou, Honglian Li.
